# Scientific

**Published:** 1889-07

**Authors:** 


### SCIENTIFIC ITEMS.

**Electric Watches and Clocks.**—M. L. Hussey, of Menlo Park, N. J., has secured a patent for a watch to run by electricity. Mr. Hussey has been 11 years at work on his inventions, and has secured patents on 13 appliances necessary in the manufacture of his clocks and watches. There are four of these, including a marine clock. The peculiarity about them is the gravity movement, which, aided by a small electric current, the pendulums of the clocks and the large balance wheels of the watches. The battery is enclosed in the watch-case, and with it the time-piece will run for a whole year without any attention. In time it is expected that five-year watches and clocks can be manufactured. The new watches and clocks contain only one-third as many parts as the ordinary instrument.—*Pop. Science News.*

**Automatic candles.**—A new candle has been been brought out, which extinguishes itself in an hour. This it does by means of a tiny extinguisher of tin, which is fastened in the wax by wires, and which effectually performs its task. It is only necessary to remove this diminutive extinguisher when its work is done, and the candle is again ready to burn another hour.—*Ib.*

**A new Antidote** for morphine is recommended in the *Internationale Klinische Rundschau*, by Prof. Arpao Bokai. This is picrotoxine, which he says, exerts an antagonistic action to morphine on the respiratory centres; for, while morphine tends to paralyze these centres, picrotoxine exerts a powerful stimulating effect. Prof. Bokai further suggests that the previous administration of a small dose of picrotoxine might reduce the danger of asphyxia in chloroform narcosis.—*Ib*

**Toxicity of the Blood of Salt Water Eels.**—Massa an Italian *savant*, has conducted certain experiments which seem to have established the fact that the blood of salt-water eels is possessed of the poisonous properties of the venom of snakes, and they only need the addition of fangs to render them equally dangerous to life. The blood of these sea-eels experimented with killed animals in the same way as the venom of snakes—viz : by paralyzing the respiratory organs. Massa holds that medicaments are of no avail, the only chance being tracheotomy and the artificial pumping of air into the lungs. Might not this knowledge be turned to some account in the treatment of cases of snake-bite ?—*Lancet.*

**Origin of the Weather.**—Mr. B. G. Jenkins, F. R. A. S., has propounded an astronomical weather law, which, if it should be verified by observation, will certainly prove to be one of the most important additions to our knowledge that this century has witnessed. The new theory proceeds upon the asumption that weather is for the most part dependent upon what may be termed atmospheric tides. That such tides exist is certain, for the same causes which produce tides in the ocean are acting upon the vast areal sea which surrounds the earth, and must produce in it corresponding results. Looking down upon the upper surface of the aqueous ocean, we are naturally led to observe the tides, which are, indeed, its most obvious phenomena. But being immersed in the aerial ocean, and dwellers upon its bottom, we have not the same facilities for observing its tides, and, indeed, can know nothing about them save by inference. When, however, we set ourselves to draw inferences, there is much that can be done in that direction. Long practice in computing ocean tides has made our physicians thoroughly familiar with their causes and the method of calculating them. Mr. Jenkins has done something of the same sort for the atmospheric tides. He has computed the disturbing effect of the moon and planets upon that regular succession of tidal phases which would result from the earth's motion round the sun if the sun and the earth were the only members of the solar system, and he has thus obtained a curve representing deviations from mean weather, which, he says, compares astonishingly well with the records of some observatories to which he has had access. He cites the case of Jamaica, to which he sent out a lunar chart prepared according to his theory. "On it," he says, "occur the twenty-six crests of the lunar curve, and the twenty-six corresponding crests in the barometric curve of Jamaica." It may be that the moon is destined some day to take by scientific authority the rank as presiding deity of the weather which popular tradition has long assigned to her ; though it seems strange that such complicated phenomena as are presented by the variations of weather in our own climate, for instance, should be capable of receiving so simple an explanation. Be that as it may, it is at ieast time that scientific meteorology made a distinct advance ; and, until some comprehensive and far-reaching law is securely demonstrated, every attempt, even though perchance unsuccessful to reach it, will awaken interest and command attention—*Lancet.*

